# Nasolabial flap reconstruction in oral cancer

**DOI:** 10.1186/1477-7819-10-227

**Published:** 2012-10-30

**Authors:** Seema Singh, Rajesh Kumar Singh, Manoj Pandey

**Affiliations:** 1 Department of Surgical Oncology, Institute of Medical Sciences, Banaras Hindu University, Varanasi, 221005, India

## Abstract

**Background:**

The nasolabial flap is a simple flap used for reconstructing small intraoral defects created after the excision of malignant tumors.

**Methods:**

A retrospective analysis of 26 cases of oral cancer treated with primary excision and nasolabial flap reconstruction was carried out. In 22 cases, the excision was combined with neck dissection and facial artery ligation.

**Results:**

Good cosmetic and functional results were obtained in almost all cases. Wound dehiscence developed in three patients, while one patient developed a persistent orocutaneous fistula. Disease recurrence occurred in one patient.

**Conclusions:**

The nasolabial flap is a good flap for the reconstruction of small oral defects after excision of primary tumors and results in good overall cosmetic and functional outcome.

## Background

Several methods described for reconstructing oral defects use either pedicled or free flaps. The pectoralis major flap, a pedicled flap, is commonly used for this purpose; however, this flap is bulky and is associated with considerable donor site morbidity. Likewise, the radial forearm free flap has also become a preferable reconstruction method. It offers a large surface of thin, pliable skin that allows for complex reconstruction, but unfortunately donor site morbidity rates are quite high, for example, through delayed wound healing and exposure of tendons. The need of microsurgical expertise is a major disadvantage [1]. This makes nasolabial flaps ideal for reconstruction of small intraoral defects. The nasolabial flap is a very simple flap used for reconstruction of intraoral defects in the floor of the mouth [2,3], the tongue, cheek, commissures [4], nose tip, nasal ala, and lower eyelids [5]. The nasolabial flap may be superiorly or inferiorly based. An inferiorly based flap is useful in reconstruction of the lip, oral commissure, and anterior aspect of the floor of the mouth, while superiorly based flaps are utilized for reconstruction of the ala and tip of the nose, and the lower eyelids and cheeks. The choice of pedicle is based on the site of the defect and any need for rotation or advancement of tissue to the site of the defect [5]. The flap may be thick or thin, depending on the requirement of the defect and the thickness of the donor tissues. Intraoral reconstruction with a nasolabial flap is a simple and fast procedure with minimum donor defect and complications. This article reviews our experience with nasolabial flaps in the reconstruction of intraoral defects.

## Methods

Between 2006 and 2010, 26 patients with oral cancer underwent reconstruction of oral defects using nasolabial flaps. A primary tumor was located in the buccal mucosa in 11 patients, the alveolus in 4 patients, the tip of the tongue in 4 patients, and the commissure and lip in 7 patients. Data were collected from the patients’ operating records and were retrospectively analyzed. Being a retrospective study, this study was exempt from the Institutional Review Board; however, each participant gave written informed consent to use data and photographs for publication.

### Anatomical considerations

A unilateral nasolabial flap can cover a defect of 2 to 3 cm, whereas a bilateral flap is sufficient for a defect 5 × 5 cm. The nasolabial flap is an axial flap but may be utilized as a random flap [4]. The flap receives its blood supply from the angular artery (a branch of the facial artery), the infraorbital artery, and the transverse facial artery [6]. This rich vascular anastomosis between all the feeding vessels makes it an ideal and versatile flap for reconstruction of the anterior floor of mouth, lips, and nose tip; hence, superiorly, inferiorly, lateral, or medial based flaps can be raised [5]. The nasolabial flap can also be used as an interpolation flap in either a single or a staged technique. Disadvantages of the nasolabial flap are that there is a limited amount of tissue available, the reconstruction may lead to asymmetry, and a ‘pincushioning’ effect of the cheek can occur when the flap is used for intraoral reconstruction.

### Technique

**Figure 1:**
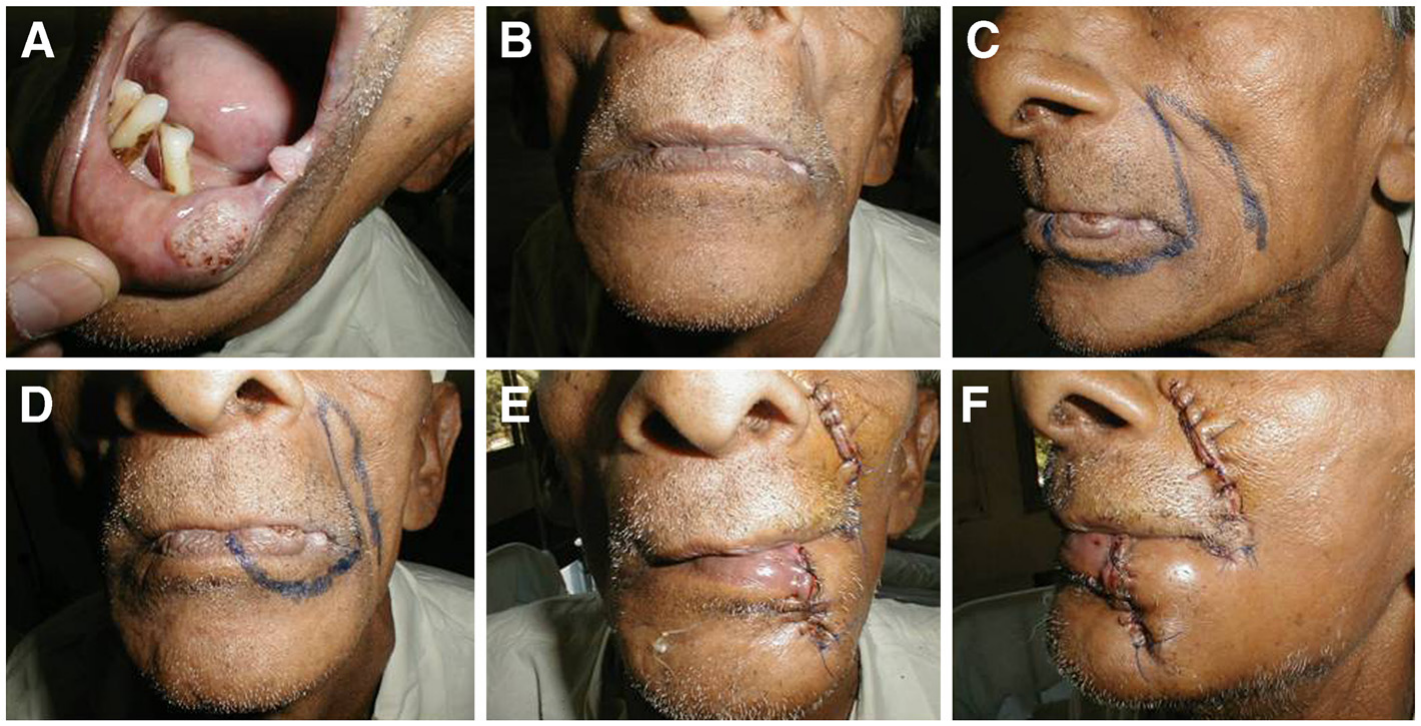
**Clinical photographs showing surgical procedure for inserting nasolabial flap.** (**A**) Two discrete lesions on the lower lip and commissure. (**B**) Front view of patient with mouth closed. (**C**) Lateral profile, showing incision. (**D**) Front view of the incision. (**E**) Front view after completion of surgery and insertion of flap. (**F**) Lateral profile after completion of surgery and insertion of flap.

The flaps are elevated directly under vision; the plane is deep to the subcutaneous tissue and superficial to the underlying muscles [7]. During dissection, the facial artery, submental artery, and external jugular vein are ligated if the neck dissection is combined with the resection of a primary tumor in a clinically node-positive neck. For all of our reconstructions, inferiorly based flaps were utilized (Figure 1). The tip of the flap was extended to a point approximately 15 mm distal to the medial canthus, while the width depended upon the width of the defect. If the facial artery was preserved, a width to length ratio of 1:3 was maintained. In cases where the facial artery was ligated, a ratio of 1:2 was maintained. After the flap was raised to the desired extent, it was rotated inwards and insetted using 4/0 Prolene® sutures. The mucosal part of the flap was sutured using 3/0 Monosyn®. When used for commissural defects, a V-Y commmissuroplasty was added as a second-stage procedure.

### Technique of nasolabial flap insetting using a tunnel

**Figure 2:**
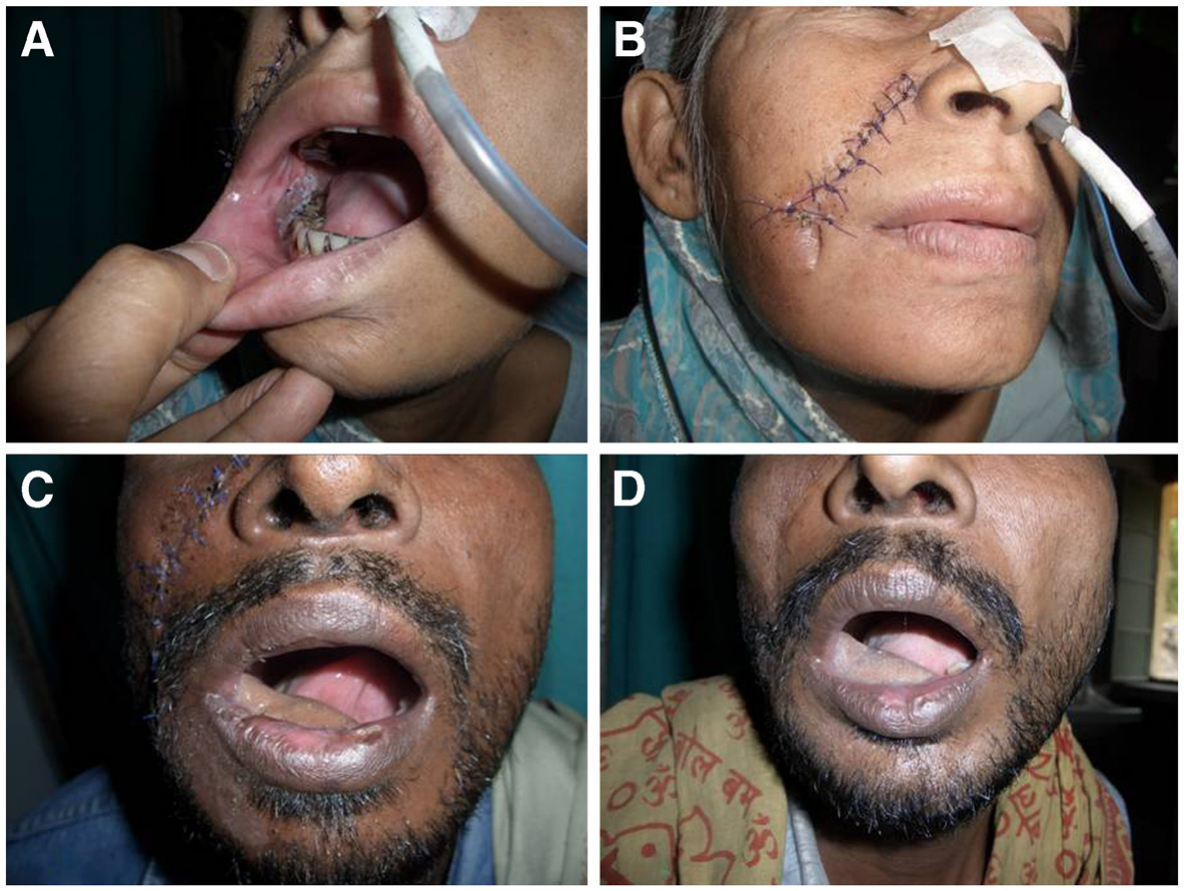
**Use of nasolabial tunnel flap.** (**A**) Intraoral view, showing flap inserted on lower alveolus. (**B**) Frontal view of same patient, showing incision and tunnel. (**C**) Postoperative view, showing flap inserted on anterior alveolus. (**D**) Late postoperative view, showing flap.

For reconstruction of the buccal mucosa, lower alveolus, tongue, or floor of the mouth where no incision was made on the lips, the flap was insetted using a buccal tunnel [8]. After 3 weeks, the flap was divided and the tunnel was closed (Figure 2).

## Results

### Patient characteristics

Of 26 patients, 22 were men and 4 women. The site of the primary tumor was the buccal mucosa in 11 patients, the tongue in four patients, the lip with commissure involvement in seven patients, and the lower alveolus in four patients.

All the patients had T2 or T3 disease with N0/N1 status on clinical examination and computed tomography and none of them received neoadjuvant radiation. Excision of the primary tumor was combined with neck dissection in 22 cases. In all 22 patients, the facial artery was dissected and preserved. In 15 cases this was achieved by intraoral excision, otherwise it was achieved through lip split. Only seven patients received postoperative adjuvant radiotherapy. Follow-up ranged from 1 year to 6 years, and no patient was lost to follow-up.

### Outcome

**Figure 3:**
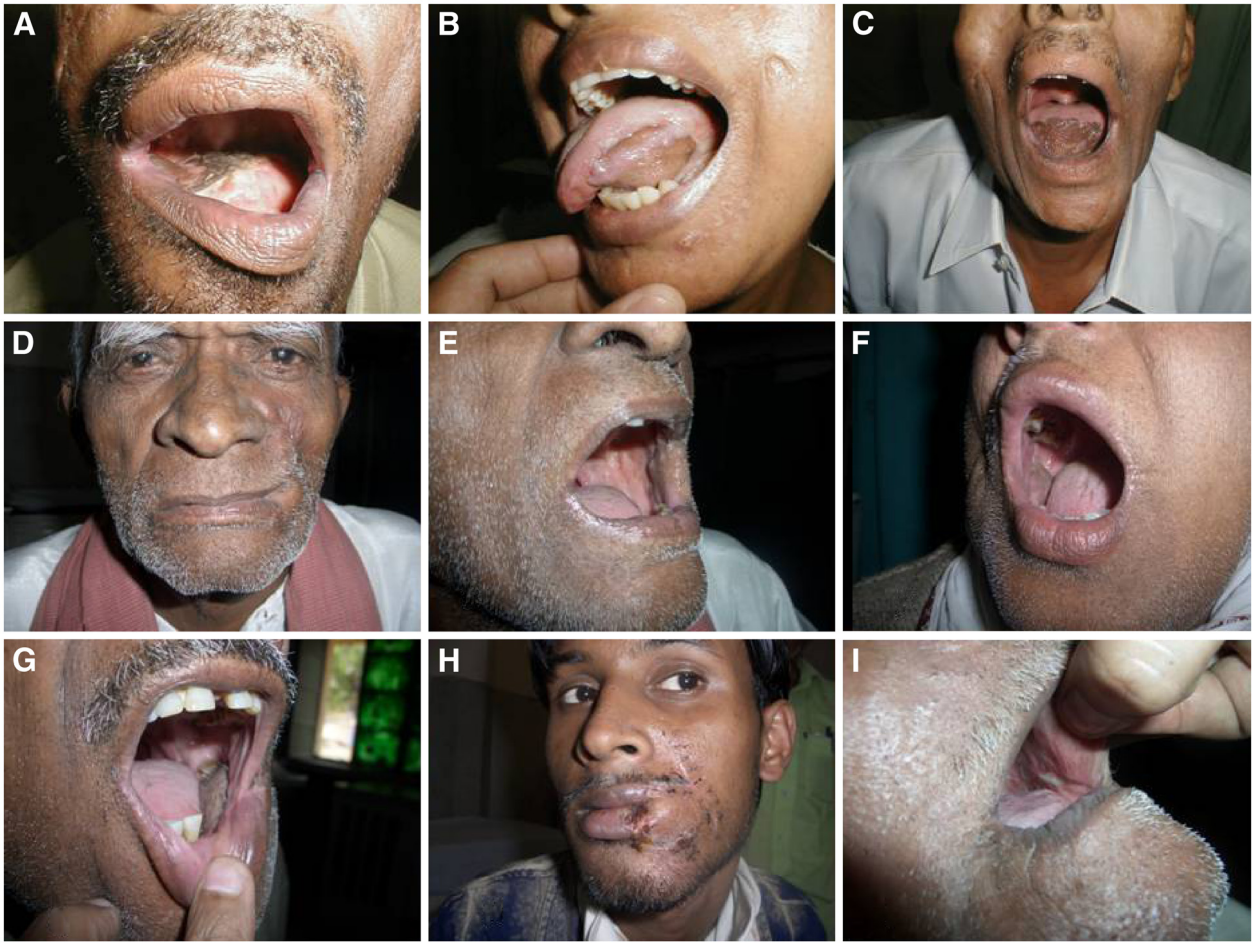
**Late postoperative clinical photographs during follow-up, showing use of nasolabial flap.** (**A**) Dorsum tongue. (**B**) Lateral tongue. (**C**) Tip of tongue. (**D**) Frontal view for reconstruction of commissure with mouth closed. (**E**) Mouth open, showing flap on commissure and buccal mucosa. (**F**) Buccal mucosa. (**G**) Bucco-gingival sulcus. (**H**) Full-thickness excision of commissure with both lips. (**I**) Buccal mucosa, showing healing after flap loss.

The cosmetic and function results were good in nearly all the patients (Figure 3). Three patients developed wound dehiscence and one developed a leak (an orocutaneous fistula). Apart from these, one patient developed wound infection requiring prolonged nasogastric feeding and antibiotic administration. Only one patient of the 26 developed recurrence. The final outcome was good in all cases, except one patient, who developed recurrence and one patient, who developed an orocutaneous fistula that required secondary closure. None of these developed trismus. No nodal failure was encountered. After the flap was healed, all the patients with T3 lesion received radiotherapy to primary and neck.

## Discussion

The versatility and usefulness of the nasolabial flap is well known [9]. The flap has a good vascular supply; hence, survival is high [10]. An abundant blood supply allows for a length to breadth ratio of 3:1. The flap is good for small and intermediate (T1 to T3) intraoral defects. The blood supply of the nasolabial flap is attributed mainly to the facial artery. However, this artery was ligated in the neck dissection in the some of our cases without any adverse effect on the viability of the flap, indicating that it may not be the facial artery but is more probably the rich subdermal plexus that supplies the skin flap [11]. The fact that this flap withstands radiotherapy signifies its excellent vascularity.

The disadvantage of this method of reconstruction is the need for a second-stage procedure in some of the cases, where a buccal tunnel is used for insetting the flap or a second-stage commissural correction is required. These procedures are minor and so can be done under local anaesthesia.

There may be other problems, such as cheek biting or a bulky base of the flap passing over the alveolus, causing problems in those wearing dentures, especially when the flap is used to repair alveolar defects (Figure 2). Dental implants may provide a good solution to this problem. Possible post-reconstruction outcomes are flap necrosis due to hematoma, infection, or tension on the suture line, where further surgery may be required. Although rare, one may encounter wound complications and partial or total reconstruction failure owing to insufficient arterial flow or venous drainage [12]. Flap survival depends on the early recognition of flap compromise, such as ischemia and necrosis. Smoking is also associated with an increased risk of flap failure because smoking has deleterious effects on flap survival by aggravating hypoxemia and vasoconstriction. Hematoma may result from inadequate hemostasis and drug-induced coagulopathy, hence medications inducing coagulopathy, for example, acetylsalicylic acid and non-steroidal anti-inflammatory drugs and vitamin E, should be avoided at least 2 weeks before and 1 week after surgery. Hematoma formation may reduce tissue perfusion and can lead to ischemia and necrosis by inducing vasospasm and stretching of the subdermal plexus or by separating the flap from its recipient bed [5].

Congestion is the most common problem associated with facial flaps. Venous congestion can lead to arterial compromise and flap necrosis. Infection can also complicate flap healing. The postoperative wound infection rate is 2.8% for facial surgery, with higher rates in facial reconstruction using local flaps. The use of flaps for reconstruction may interfere with the normal sensation and neurological afferent control that provides sensory guidance to speech and swallowing. Furthermore, especially in men, if a flap is taken from hair-bearing skin to reconstruct a surgical defect, then that area of tissue will continue to grow hair. This can be prevented by outlining the flap. It can also be seen that postoperative radiotherapy may decrease the growth of hair and ultimately lead to mucosalization of the flaps. There may also be a pincushioning effect around the nasolabial folds, which could be avoided by using a rhomboid design [13]. An ipsilateral nasolabial flap can cover small defects up to 2 cm but if a larger defect of size approximately 5 × 5cm or more is to be reconstructed, a bilateral nasolabial flap can be utilized successfully.

## Conclusion

The nasolabial flap is versatile for covering or reconstructing small or medium-sized defects of the oral cavity in selected patients. However, this type of reconstruction is not particularly suitable when teeth are present in the area to be reconstructed and biting on the pedicle may even damage the skin. As even small defects require reconstruction, the nasolabial flap has proven to be a useful and reliable alternative without causing much morbidity to the donor site.

## Competing interests

The authors declare that they have no competing interests.

## Authors’ contributions

SS: Did the literature search and prepared the manuscript. RS: collected and analysed the data and helped in preparation of manuscript. MP: overall supervision, concept and design, preparation of final manuscript. All authors read and approved the final manuscript.
